# Investigating the Shared Mechanisms of Endocrine-Disrupting Chemicals in Urogenital Tumors

**DOI:** 10.3390/biology15120946

**Published:** 2026-06-17

**Authors:** Cundong Liu, Shenghao Wu, Ranran Zhou, Shan Xiao, Cheng Yang

**Affiliations:** 1 Department of Developmental Biology, School of Basic Medical Sciences, Southern Medical University, Guangzhou 510630, China; 2 Department of Urology, The Third Affiliated Hospital, Southern Medical University, Guangzhou 510630, China; wushenghao1225@outlook.com (S.W.); zhouranran1995@163.com (R.Z.); 3 The Third Clinical School, Southern Medical University, Guangzhou 510630, China

**Keywords:** endocrine-disrupting chemical, bladder cancer, renal cell carcinoma, prostate cancer, testicular germ cell tumor, shared targets

## Abstract

Endocrine-disrupting chemicals (EDCs) are important environmental risk factors for urogenital malignancies, but the shared molecular mechanisms underlying their carcinogenic effects remain poorly understood. Here, we systematically investigated the common pro-tumorigenic mechanisms of 12 prevalent EDCs, including anthracene, benzo[a]pyrene (BaP), bisphenol A, clofenotane, di(2-ethylhexyl) phthalate, diazinon, dibutyl phthalate, glyphosate, malathion, perfluorooctanoic acid, polychlorinated biphenyls, and triclosan, across four urogenital cancers, including bladder cancer (BLCA), renal cell carcinoma (RCC), prostate adenocarcinoma (PRAD), and testicular germ cell tumor (TGCT). By integrating network toxicology and protein–protein interaction analysis, we identified shared hub targets linking EDC exposure to tumor progression. EGFR and CASP3 were identified as core targets in BLCA, EGFR and CASP9 in RCC, and CASP3, ESR1, and EGFR in PRAD, whereas KIT emerged as a broadly relevant target in TGCT. Molecular docking and molecular dynamics simulations supported the stable binding of EDCs to these targets. Among the predicted interactions, BaP showed strong binding affinity for CASP9 (ΔG = −9.8 kcal/mol) and was therefore selected for experimental validation. Analysis of TCGA data showed that elevated CASP9 expression was significantly associated with poorer overall survival in patients with RCC. In 786-O and ACHN cells, chronic exposure to an environmentally relevant concentration of BaP significantly increased CASP9 protein stability without altering its mRNA expression, suggesting post-transcriptional regulation. Collectively, these findings identify shared molecular targets of EDCs across urogenital cancers and provide new mechanistic insight into EDC-driven tumor progression, prioritizing potential biomarkers and therapeutic targets for environmentally related malignancies.

## Simple Summary

This study systematically investigates the shared molecular mechanisms through which prevalent endocrine-disrupting chemicals (EDCs) may contribute to the pathogenesis of four major urogenital cancers: bladder cancer (BLCA), renal cell carcinoma (RCC), prostate adenocarcinoma (PRAD), and testicular germ cell tumor (TGCT). Using an integrated computational framework combining network toxicology, protein–protein interaction analysis, molecular docking, and dynamics simulations, the research identifies common hub protein targets linking exposure to 12 EDCs with tumor progression in a cancer-type-specific manner. Key shared targets include EGFR and CASP3 in BLCA, EGFR and CASP9 in RCC, CASP3, ESR1, and EGFR in PRAD, and KIT in TGCT. The benzo[a]pyrene (BaP)–CASP9 interaction, predicted to have high binding affinity, was selected for experimental validation. Results show that chronic exposure to an environmentally relevant concentration of BaP post-transcriptionally increases CASP9 protein stability in multiple urogenital cancer cell lines. These findings delineate converging molecular pathways for EDCs across different urogenital malignancies and highlight potential biomarkers and therapeutic targets for environmentally associated cancers.

## 1. Introduction

Urogenital malignancies such as bladder cancer (BLCA), renal cell carcinoma (RCC), prostate cancer (PRAD), and testicular germ cell tumor (TGCT) remain a substantial global health burden because of their high incidence and mortality rates [1,2]. Aging and inherited susceptibility contribute substantially to risk, but do not fully explain the heterogeneity in disease incidence across individuals and populations. A considerable proportion of cases may arise from environmental exposures [3]. Among these, endocrine-disrupting chemicals (EDCs) represent a broad class of environmental contaminants originating from plastics, pesticides, personal care products, and industrial waste, all of which can interfere with hormonal balance [4,5]. Epidemiological studies have shown that chronic exposure to EDCs increases the risk of several hormone-related cancers including malignancies of the urogenital system [6,7,8,9,10,11]. Mechanistically, these compounds promote carcinogenesis through intertwined genomic and non-genomic signaling pathways that disrupt core cellular functions such as proliferation, apoptosis, and differentiation [6,12].

Despite epidemiological associations, the molecular mechanisms by which EDCs initiate and promote urogenital tumors remain unclear. Moreover, much of the early research focused on examining the biological activity and mechanistic effects of a single EDC. Although informative, this approach leaves a significant gap. Real-world human exposure is chronic and typically involves complex chemical mixtures, rather than a single agent [13]. In such settings, co-exposure can produce a “cocktail effect,” in which interactions among compounds lead to additive, synergistic, or cumulative toxic outcomes [7]. From a biomedical perspective, this issue is complex. Therefore, a more systematic dissection of the molecular networks shared by prevalent EDCs in urogenital malignancies is needed, both to refine environmental risk assessment and to uncover potentially actionable therapeutic targets.

Network toxicology has increasingly been applied to investigate the potential carcinogenic mechanisms of environmental chemicals, including EDCs. Previous studies using target prediction, disease-gene mapping, protein–protein interaction (PPI) networks, and pathway enrichment analyses have provided useful evidence that compounds such as bisphenol A, phthalates, polycyclic aromatic hydrocarbons, and polychlorinated biphenyls may regulate cancer-associated pathways, including PI3K-AKT, MAPK, apoptosis, oxidative stress, steroid hormone signaling, and inflammatory responses [14,15,16,17]. In addition, several pan-cancer or multi-cancer computational frameworks have attempted to identify common molecular targets of environmental toxicants across different tumor types [18,19,20,21]. However, these studies have several limitations. First, many investigations were either chemical-centered, focusing on a single EDC or a narrow class of compounds, or cancer-centered, focusing on one malignancy, thereby limiting their ability to reflect real-world co-exposure to multiple EDCs. Second, although some multi-cancer analyses have been performed, they often grouped biologically heterogeneous tumors together and did not specifically address urogenital malignancies as a clinically and developmentally related cancer spectrum. Third, most previous studies remained primarily predictive, with limited integration of molecular docking, dynamic binding stability, clinical prognostic relevance, and experimental validation. Therefore, the shared and tumor-specific molecular vulnerabilities through which prevalent EDCs may contribute to BLCA, RCC, PRAD, and TGCT remain insufficiently defined.

In this study, an integrated network toxicology framework was employed. We combined computational prediction with experimental validation to systematically map the shared molecular networks linking EDC exposure to urogenital cancer pathogenesis. Specifically, the analysis focused on 12 commonly encountered EDCs and 4 major urogenital malignancies, including BLCA, RCC, PRAD, and TGCT. Our aim was to identify candidate protein targets that might link exposure to these chemicals to tumorigenesis of distinct urogenital tumors. We further examined how these EDCs interact at the molecular level with key oncogenic and tumor-suppressive targets, as well as their consequent effects on target protein regulation. Overall, this work provides novel insights that may support the identification of biomarkers and development of therapeutic strategies for EDC-associated urogenital tumors.

## 2. Materials and Methods

### 2.1. Carcinogenicity Evaluation of EDCs

The chemical structures and SMILES sequences of 12 EDCs, comprising anthracene, benzo[a]pyrene (BaP), bisphenol A, clofenotane, di(2-ethylhexyl) phthalate (DEHP), diazinon, dibutyl phthalate, glyphosate, malathion, perfluorooctanoic acid, polychlorinated biphenyls, and triclosan [22,23,24], were sourced from PubChem (Table S1). The carcinogenic risk of these EDCs was assessed using admetSAR 3.0, ProTox 3.0, and ADMETlab 3.0.

### 2.2. Predicting EDCs’ Targets

The targets of EDCs were predicted using ChEMBL (version 34), PharmMapper (version 2017), Similarity Ensemble Approach (SEA, accessed 6 November 2025), SwissTargetPrediction (accessed 6 November 2025), and TargetNet (accessed 6 November 2025). Predictions were refined to include solely human targets with a likelihood score exceeding 0 [25]. This permissive threshold was chosen to minimize false negatives at this stage, with the understanding that subsequent rigorous bioinformatic filtering would prioritize the most biologically relevant and high-confidence central targets from this larger pool. Subsequently, duplicate entries were eliminated to generate a distinct target list for subsequent research.

### 2.3. Identifying Urogenital Tumors’ Targets

Targets related to urogenital malignancies were found by the integration of omics analysis with curated disease-gene retrieval. RNA-seq data (FPKM) for tumor tissues from TCGA, in addition to healthy tissue data from GTEx (FPKM), were acquired through the UCSC Xena platform. Following the integration and batch correction of the expression matrices utilizing the sva package in R (version 4.4.2), differential expression analysis was conducted employing the limma R package, applying thresholds of |log2FC| > 1 and a false discovery rate (FDR) <0.05. Subsequently, utilizing clinical follow-up data from the TCGA cohort, a univariate Cox regression analysis was conducted with the survival package to evaluate the correlation of these differentially expressed genes with overall survival (OS, for BLCA, RCC, and TGCT) or biochemical recurrence (BCR, for PRAD), retaining genes exhibiting significant prognostic relevance (*p* < 0.05). Disease-associated genes were concurrently obtained from the Therapeutic Target Database (TTD), GeneCards, and OMIM with specified search phrases (Table S2). In the GeneCards database, genes with a relevance score >10 were classified as disease-associated genes [26]. The ultimate target established was obtained by integrating the expression-based prognostic genes with the text-mined candidates and eliminating duplicates.

To assess the concordance across prediction platforms and the robustness of the initial target pool, we performed a sensitivity analysis. We calculated the overlap of predicted targets for each EDC across the five platforms. A core set of 117 targets (those predicted by ≥3 platforms) was identified, demonstrating good inter-platform agreement. The subsequent PPI network and hub gene analysis were performed on both the full union set and this high-confidence core set. The identified hub genes, including PTGS2, CASP3, SRC, PPARG, EGFR, ESR1, and HSP90AA1, remained consistent between the two analyses, confirming the robustness of our findings against variations in the initial target prediction threshold. Detailed results are provided in Figure S1.

### 2.4. Detecting Hub Targets Linking EDCs to Urogenital Tumors

The intersection of predicted EDC targets and disease-associated targets for each urogenital carcinoma was identified to establish a set of shared targets. A PPI network for these common targets was subsequently created utilizing the STRING database, with a confidence score threshold set at 0.4. The PPI data was analyzed in Cytoscape software (version 3.8.0) using two complementary algorithms, cytoHubba and MCODE, to find the most essential nodes within the network. Genes rated in the top 10 by degree were picked from the cytoHubba analysis [27]. Simultaneously, the MCODE analysis was employed to identify genes associated with the most densely interconnected cluster in the PPI network. The definitive set of high-confidence hub targets was established as the genes identified by both cytoHubba and MCODE investigations [28].

### 2.5. Functional Enrichment

The functional enrichment analysis of targets associated with EDCs and urogenital cancers was performed using the Metascape web tool (version 3.5), with terms having a q value < 0.05 being significant.

### 2.6. Immunohistochemistry (IHC)

IHC analysis was performed using publicly available data from the Human Protein Atlas (HPA) database. Representative IHC images and protein expression annotations for normal tissues and urogenital tumor tissues were retrieved from HPA to evaluate the expression patterns of the identified hub proteins. The HPA IHC data are generated from tissue microarrays and provide representative staining intensity and distribution patterns across different tissue types.

### 2.7. Molecular Docking

To examine the probable binding interactions between the prioritized EDCs and the identified hub protein targets at an atomic level, molecular docking was conducted. The three-dimensional crystal structures of the target proteins were obtained from the PDB, with the corresponding PDB IDs listed in Table S3. The protein structures were created by eliminating water molecules and heteroatoms, including hydrogen atoms, and assigning Gasteiger charges utilizing PyMOL (version 2.5) and AutoDockTools (version 1.5.7). The three-dimensional chemical structures of the 12 EDCs were acquired in SDF format from PubChem and subsequently translated to PDBQT format following energy minimization with Open Babel. Molecular docking was performed via AutoDock Vina (version 1.1.2). The grid box was centered on the active site or potential binding pocket of each target protein, with dimensions adequate to encompass the ligand. The docking parameters were configured to default settings, and the exhaustiveness was elevated to 24 to guarantee comprehensive sampling of the conformational space. For each ligand–protein pair, the docking simulation was conducted 10 times, and the conformation exhibiting the most advantageous binding affinity was chosen for subsequent molecular dynamics simulation. The binding conformations and distinct molecular interactions were observed and examined with PyMOL and LigPlot+ (version 2.3) [29].

### 2.8. Molecular Dynamics Simulation

Molecular dynamics simulations were used to assess the stability of the EDC–target complexes predicted by molecular docking and to compute more precise binding free energies. The highest-ranked docking pose for critical complexes was utilized as the initial structure. The system was constructed utilizing the tleap module of the AmberTools22 package. The protein was characterized using the ff19SB force field, whereas the ligands were parameterized with the generic AMBER force field (GAFF2) employing AM1-BCC charges. Each complex was solubilized in a truncated octahedral box of TIP3P water molecules, maintaining a minimum gap of 12 Å between the solute and the box boundary. Counterions were introduced to equilibrate the system’s charge. Energy minimization was conducted in two phases: initially constraining the solute, followed by permitting the entire system to equilibrate. The system was incrementally heated from 0 K to 310 K over 100 ps within an NVT ensemble, applying harmonic restraints on the solute, followed by a 500 ps equilibration in an NPT ensemble (1 atm) with progressively diminished restraints. Production molecular dynamics simulation was conducted for 100 nanoseconds under an NPT ensemble (310 K, 1 atm) utilizing the pmemd.cuda module of AMBER22. The particle mesh Ewald approach was employed to address long-range electrostatic interactions, with a 10 Å threshold implemented for non-bonded interactions. Root-mean-square deviation (RMSD) analysis was conducted with CPPTRAJ. The binding free energy of the last stable complex was computed utilizing the Molecular Mechanics/Generalized Born Surface Area (MM/GBSA) method on 500 uniformly sampled frames from the last 50 nanoseconds of the trajectory [30].

### 2.9. Cell Culture

The human RCC lines 786-O and ACHN were acquired from the American Type Culture Collection (ATCC, Manassas, VA, USA). Cells were grown in RPMI-1640 media (Gibco, Grand Island, NY, USA). The human BLCA cell line T24, PRAD cell line LNCaP, and TGCT cell line Tcam-2 were also obtained from ATCC. T24 and LNCaP cells were cultured in RPMI-1640 medium, and Tcam-2 cells were cultured in DMEM medium. All medium was supplemented with 10% FBS (Gibco, Grand Island, NY, USA) and 1% penicillin–streptomycin (Gibco, Grand Island, NY, USA). All cell lines were sustained in a humidified incubator at 37 °C with 5% CO_2_ concentration.

Cells were treated with BaP at 100 nM for 7 days, with medium replaced every 24 h. The vehicle control group received 0.1% DMSO. Cell viability was assessed using the trypan blue staining, and no significant increase in mortality was observed under the selected treatment condition.

### 2.10. Chemicals

BaP (purity > 96%, Catalog No. B1760) was acquired from Sigma-Aldrich (St. Louis, MO, USA). A 10 mM stock solution was created by dissolving BaP in high-purity dimethyl sulfoxide (DMSO, Sigma-Aldrich, St. Louis, MO, USA) [31]. The stock solution was aliquoted, shielded from light, and preserved at −20 °C. A fresh working solution was prepared for all in vitro treatments by diluting the stock solution in full cell culture medium to a final concentration of 0.1 μM (100 nM) immediately before use.

### 2.11. Protein Stability Assay

A cycloheximide (CHX) test was conducted to assess the impact of BaP on CASP9 protein stability. Cells were inoculated in 6-well plates and permitted to adhere overnight. Cells were subsequently exposed to 0.1 µM BaP or an equal volume of DMSO (Merck, St. Louis, MO, USA) for 7 days. This dose is biologically relevant, as previous studies have reported that the concentration of free BaP in human serum ranges approximately from 0.5 nM to 147.3 nM [32]. Following pretreatment, we replaced the medium with fresh medium containing 50 µg/mL of the protein synthesis inhibitor CHX (MCE, Hefei, China). Whole-cell lysates were harvested at the indicated time points, namely 0, 6, and 12 h after CHX administration. Protein concentrations were measured with a BCA kit (Beyotime, Shanghai, China). For immunoblotting, equal amounts of protein (20 µg) from each sample were resolved by sodium dodecyl sulfate–polyacrylamide gel electrophoresis (SDS-PAGE) and transferred to polyvinylidene fluoride (PVDF) membranes. The membranes were then blocked and incubated overnight at 4 °C with primary antibodies against CASP9 (1:2000, Wuhan, China) and β-actin (1:10,000, Abclonal, Woburn, MA, USA). After incubation with the HRP-conjugated secondary antibodies (1:2000, ABclonal), protein bands were detected using an ECL kit (Beyotime, Shanghai, China). Band intensities were quantified with ImageJ software (version 1.54p, National Institutes of Health, Bethesda, MD, USA).

### 2.12. mRNA Stability Assay

To assess whether BaP regulates CASP9 at the post-transcriptional level, we evaluated CASP9 mRNA stability using an actinomycin D (ActD) assay. Briefly, cells were seeded into 6-well plates and pretreated with 0.1 µM BaP or DMSO for 7 days. We then blocked transcription by adding ActD (5 µg/mL, MCE, Hefei, China) to the culture medium. Total RNA was subsequently collected at 0, 3, 6, and 9 h after ActD exposure using TRIzol reagent (YEASEN, Shanghai, China) in accordance with the manufacturer’s instructions. RNA concentration and purity were measured on a NanoDrop spectrophotometer (Thermo Fisher Scientific, Waltham, MA, USA). Complementary DNA (cDNA) was synthesized from 1 µg total RNA using a PrimeScript RT reagent kit with gDNA Eraser (Takara, Kusatsu, Shiga Prefecture, Japan). Quantitative real-time PCR (qRT-PCR) was then performed with TB Green Premix Ex Taq II (Takara, Japan) on a QuantStudio 3 Real-Time PCR System (Applied Biosystems, Foster City, CA, USA). CASP9-specific primers and primers for the reference gene GAPDH were designed and produced by GenScript (Nanjing, China), and their sequences are listed in Table S4. Relative CASP9 mRNA levels at each time point were calculated using the 2^−ΔΔCt^ method and normalized to GAPDH.

### 2.13. Statistical Analysis

All statistical analyses were carried out using R software (version 4.4.2) or GraphPad Prism (version 9.0.0). Data are presented as the mean ± standard deviation (SD) from at least three independent experiments. For comparisons between two groups, we applied Welch’s corrected *t*-test. To examine the association between gene expression and patient survival, we used Kaplan–Meier survival analysis with the log-rank test through the survival R package. The optimal cut-off value for survival analysis was detected using the X-tile software (version 3.6.1). For CHX chase assays, CASP9 protein levels (normalized to β-actin and to the 0 h time point) were compared between BaP-treated and DMSO control groups using a linear mixed-effects model with time as a repeated measure. This global analysis tested for overall differences in protein degradation kinetics across the 0, 6, and 12 h time points. A *p* value < 0.05 was considered statistically significant. The website links of the public bases used in this study are provided in Table S5.

## 3. Results

### 3.1. Evaluating the Carcinogenicity of EDCs

**Figure 1:**
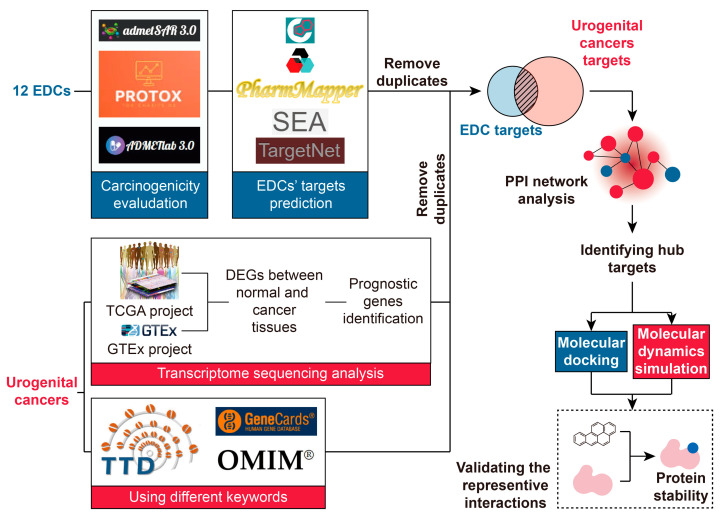
The workflow of this study.

**Figure 2:**
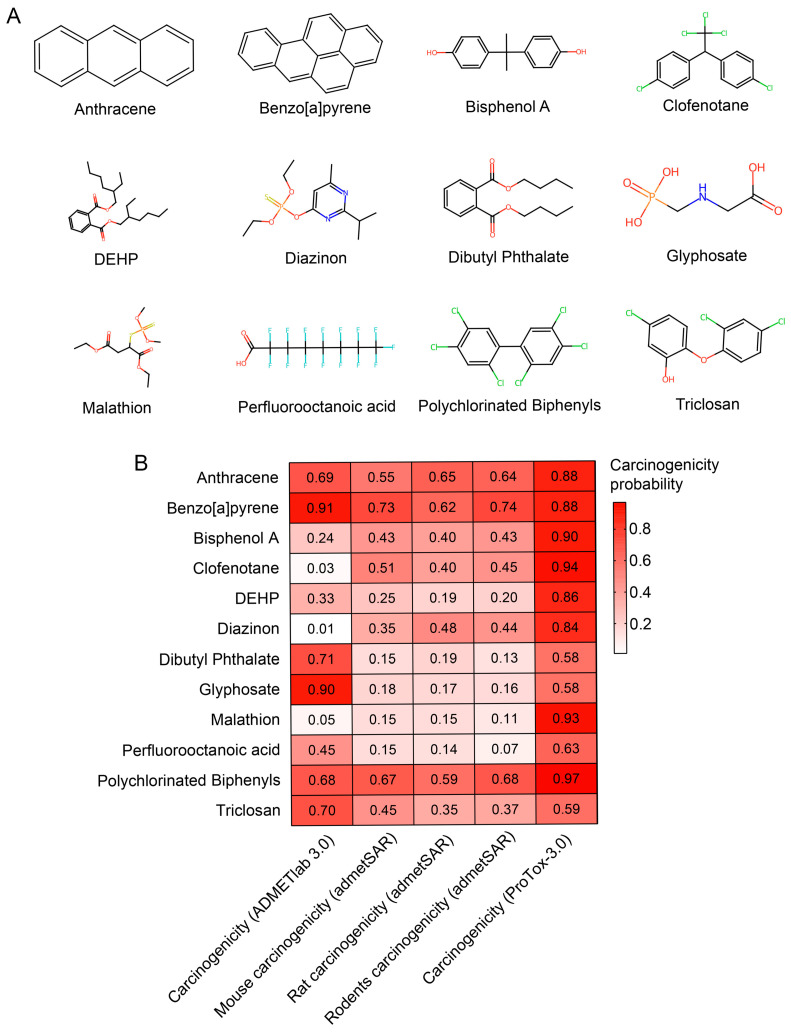
The chemical structures (**A**) and predicted carcinogenic risk (**B**) of the 12 EDCs. EDC, endocrine-disrupting chemicals.

Figure 1 summarizes the full workflow of our study. We first examined the carcinogenic potential of the 12 EDCs selected for this work. The chemical structures of the 12 DECs were obtained from PubChem (Figure 2A). For this purpose, we relied on three independent computational prediction platforms: admetSAR 3.0, ProTox 3.0, and ADMETlab 3.0. Notably, several compounds, such as anthracene, BaP, and polychlorinated biphenyls, were identified as carcinogenic by all three approaches, with each yielding a calculated carcinogenicity probability above 0.5 (Figure 2B). More importantly, on closer inspection, all 12 EDCs showed a carcinogenic probability exceeding 0.6 in at least one algorithm (Figure 2B), further reinforcing the strong link between these EDCs and tumorigenic potential.

### 3.2. EGFR and CASP3 Were Identified as Shared Targets in BLCA

**Figure 3:**
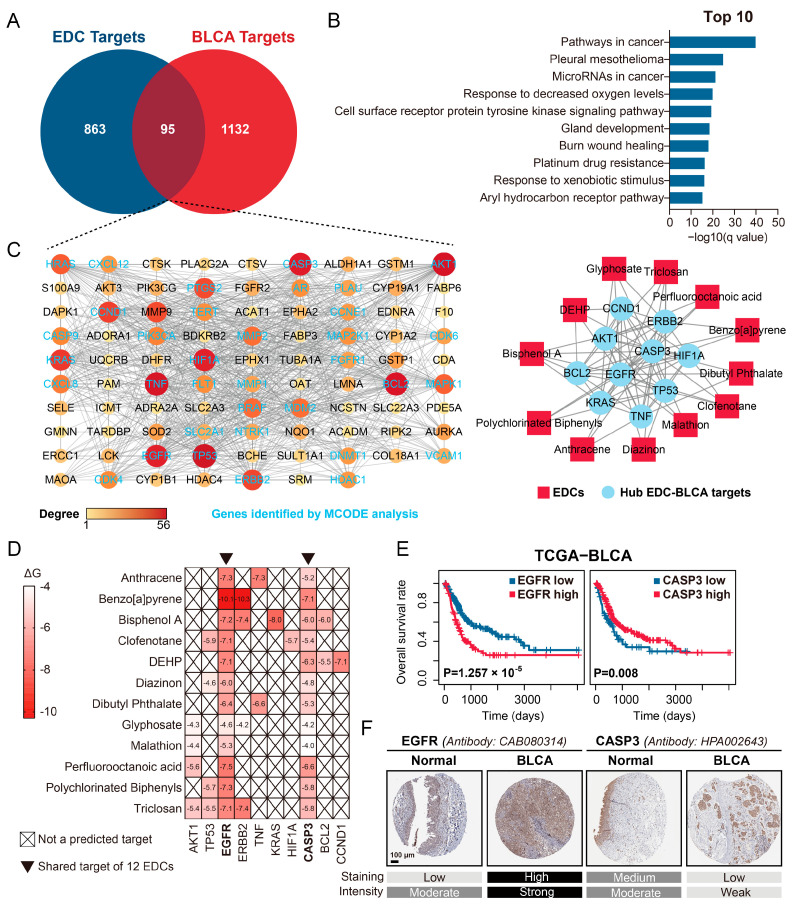
The shared targets of EDCs in BLCA. (**A**) 95 proteins were identified as the potential EDC-BLCA targets. (**B**) Functional enrichment analysis of the 95 EDC-BLCA targets performed using Metascape. (**C**) The PPI network analysis identified 10 hub genes, and EGFR and CASP3 served as shared targets of all 12 EDCs in BLCA. (**D**) Molecular docking and molecular dynamics simulation indicated the binding affinity of the EDCs with their targets. (**E**) The prognosis value of EGFR and CASP3 in the TCGA-BLCA cohort; the optimal cut-off values were detected with the X-tile software. (**F**) IHC analyses indicated the expression levels of EGFR and CASP3 in normal bladder and BLCA tissues. BLCA, bladder cancer; PPI, protein–protein interaction; EGFR, epidermal growth factor receptor; CASP3, caspase 3; TCGA, The Cancer Genome Atlas; IHC, immunohistochemistry.

We adopted an integrated computational approach to identify protein targets that may underline the effects of EDCs in BLCA. By querying five predictive platforms, including ChEMBL, PharmMapper, SEA, SwissTargetPrediction, and TargetNet, we retrieved 958 human protein targets for the 12 EDCs. At the same time, we collected 1227 BLCA-associated targets by integrating prognostic differentially expressed genes from the TCGA-BLCA and GTEx cohorts with text-mined candidates from disease-related databases. When we compared these two datasets, 95 overlapping targets were identified, which points to potential molecular connections between EDC exposure and BLCA pathogenesis (Figure 3A). Functional enrichment analysis further indicated that these 95 common targets were strongly linked to cancer-related pathways, particularly the hypoxic response and resistance to platinum-based therapy (Figure 3B). We next built a PPI network based on the shared targets and, after analyzing it with the cytoHubba and MCODE plugins in Cytoscape, identified 10 hub genes: AKT1, TP53, EGFR, ERBB2, TNF, KRAS, HIF1A, CASP3, BCL2, and CCND1 (Figure 3C). Notably, EGFR and CASP3 appeared as common targets of all 12 EDCs (Figure 3C). To examine binding behavior more closely, we performed molecular docking and molecular dynamics simulation analyses between the 12 EDCs and the selected hub targets. The results showed that EGFR and CASP3 consistently exhibited strong binding affinities, with all ΔG ≤ −4.0 kcal/mol (Figure 3D). We next assessed the pathogenic relevance of EGFR and CASP3 through clinical correlation and protein expression analyses. Kaplan–Meier survival analysis of the TCGA-BLCA cohort revealed that higher EGFR expression or lower CASP3 expression was significantly associated with worse OS in patients (both *p* < 0.01, Figure 3E). Consistent with these findings, IHC confirmed that EGFR protein expression was increased, whereas CASP3 expression was decreased, in BLCA tumor tissues compared with normal bladder tissues (Figure 3F). Taken together, these findings map a target landscape for EDCs in BLCA and computationally identify EGFR and CASP3 as key shared molecular targets.

### 3.3. EGFR and CASP9 Were Identified as Shared Targets in RCC

We compiled 1816 RCC-associated targets by integrating prognostically relevant differentially expressed genes from the TCGA-RCC cohort, including TCGA-KIRC, TCGA-KICH, and TCGA-KIRP datasets, together with GTEx data and text-mined candidates from disease databases. When this set was intersected with the 958 predicted EDC targets described above, 138 shared targets emerged, suggesting a possible link between EDC exposure and RCC pathogenesis (Figure S2A). Functional enrichment analysis showed that these 138 shared targets were strongly associated with cancer-related pathways, particularly responses to hypoxia and external stimuli (Figure S2B). PPI network analysis further highlighted nine hub genes: AKT1, EGFR, HIF1A, PTGS2, BCL2, CCND1, KDR, ICAM1, and CASP9 (Figure S2C). Notably, EGFR and CASP9 appeared as common targets across all 12 examined EDCs (Figure S2C). Molecular docking and molecular dynamics simulation indicated that both EGFR and CASP9 exhibited marked binding affinity with the EDCs, with all ΔG < −4.0 kcal/mol (Figure S2D). Kaplan–Meier survival analysis of the TCGA-RCC cohort showed that higher expression of EGFR or CASP9 was significantly associated with poorer OS in RCC patients (both *p* < 0.05, Figure S2E). IHC analysis likewise demonstrated that EGFR and CASP9 protein levels were markedly increased in RCC tumor tissues compared with normal kidney tissues (Figure S2F). Taken together, these findings outline the target landscape and computationally identify EGFR and CASP9 as shared molecular mediators linking EDC exposure to RCC pathogenesis.

### 3.4. CASP3, ESR1, and EGFR Were Identified as Common Targets in PRAD

We assembled 2097 PRAD-associated targets by integrating prognostically relevant differentially expressed genes from the TCGA-PRAD and GTEx cohorts with text-mined candidates from disease databases. When this set was intersected with the 958 predicted EDC targets identified earlier, 272 shared targets emerged, which may act as potential mediators of EDC effects in PRAD pathogenesis (Figure S3A). Functional enrichment analysis showed that these 272 common targets were mainly associated with general cancer pathways, the PI3K-Akt signaling pathway, and regulation of cell motility (Figure S3B). The PPI network and subsequent hub gene analysis narrowed these common targets to 10 core genes: SRC, TNF, CASP3, BCL2, HIF1A, TP53, NFKB1, AKT1, ESR1, and EGFR (Figure S3C). Among them, CASP3, ESR1, and EGFR were shared across all 12 EDCs examined (Figure S3C). Molecular docking and molecular dynamics simulation indicated that CASP3, ESR1, and EGFR each exhibited strong binding affinities with the EDCs, with all ΔG < −4.0 kcal/mol (Figure S3D). Analysis of the TCGA-PRAD cohort further supported their clinical relevance, showing that higher expression of CASP3, ESR1, or EGFR was significantly associated with an increased likelihood of BCR in patients (all *p* < 0.05, Figure S3E). In addition, IHC analysis revealed that CASP3, ESR1, and EGFR protein expression was markedly higher in PRAD tumor tissues than in normal prostate tissues (Figure S3F). Together, these findings define the EDC-related target landscape in PRAD and computationally identify CASP3, ESR1, and EGFR as key shared molecular targets linking EDC exposure to PRAD pathogenesis.

### 3.5. KIT Was Identified as a Near-Universal Target in TGCT

To clarify the molecular connections between EDC exposure and TGCT, we used the same integrative analytical approach. This yielded a collection of 405 TGCT-related targets. Intersecting this set with the 958 predicted EDC targets produced 35 shared targets, pointing to possible mediators of EDC effects in TGCT pathogenesis (Figure S4A). Functional enrichment analysis showed that these 35 common targets were significantly associated with cancer proliferative signaling, immune-related pathways, and lipid metabolism in senescent cells (Figure S4B). We then built a PPI network for these shared targets and, after hub gene analysis, identified seven hub targets: GRB2, KRAS, BRAF, TP53, KIT, HRAS, and EPHA2 (Figure S4C). KIT emerged as a common target for 11 of the 12 EDCs examined, with anthracene as the only exception (Figure S4C). Notably, KIT also maintained stable binding affinities with these 11 EDCs, with all ΔG < −4.0 kcal/mol (Figure S4D). Kaplan–Meier survival analysis of the TCGA-TGCT cohort further showed that higher KIT expression was significantly associated with better OS in TGCT patients (*p* < 0.05, Figure S4E). Consistent with this, IHC analysis demonstrated reduced KIT protein expression in TGCT tumor tissues compared with normal testicular tissues (Figure S4F). Taken together, these findings define the EDC-related target landscape in TGCT and computationally identify KIT as a key, nearly universal molecular target linking EDC exposure to TGCT pathogenesis.

### 3.6. Validation of a Representative EDC–Target Interaction: BaP Stabilizes CASP9 Protein in Multiple Urogenital Tumor Cells

**Figure 4:**
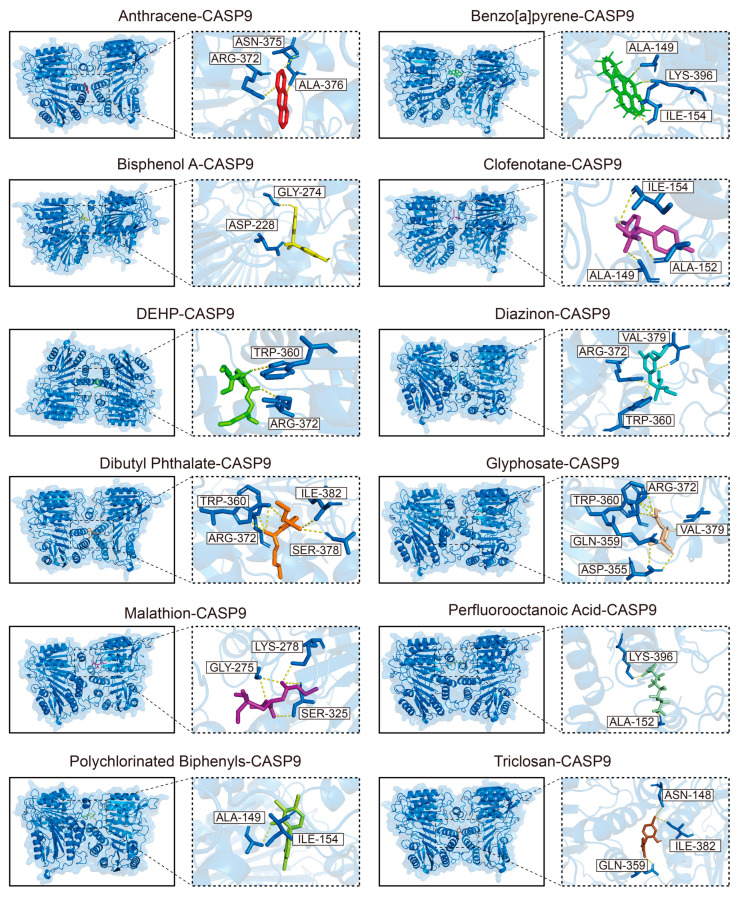
Predicting the binding poses of the 12 EDCs with CASP9 using molecular docking. Hydrogen bonds are indicated by yellow dashed lines. Blue represents the CASP9 protein, and other colors mean the EDC.

**Figure 5:**
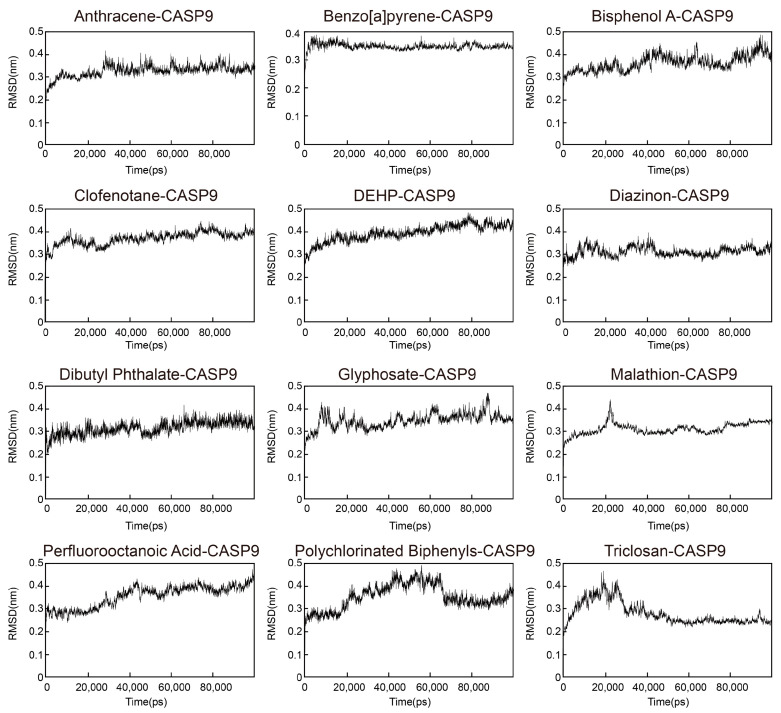
The RMSD trajectories of all 12 EDC–CASP9 complexes in molecular dynamics simulation. RMSD, root-mean-square deviation.

To verify the representative EDC–target interactions identified in this study, we carried out a series of in vitro functional assays. Among the common targets found in urogenital malignancies, we selected CASP9 for further investigation because it showed high binding affinity with all 12 EDCs (all ΔG < −5.0 kcal/mol, Figure S2D), especially in RCC. The predicted binding poses and the stability profiles of the 12 EDCs complexed with CASP9 are shown in Figure 4 and Figure 5, respectively. As the molecular dynamics simulation results showed, the RMSD trajectories of all 12 EDC-CASP9 complexes remained stable, with fluctuations staying below 0.3 nm overall (Figure 5). Among these EDCs, BaP displayed the strongest affinity for CASP9 (ΔG = −9.8 kcal/mol, Figure S2D), so we chose it for experimental validation.

**Figure 6:**
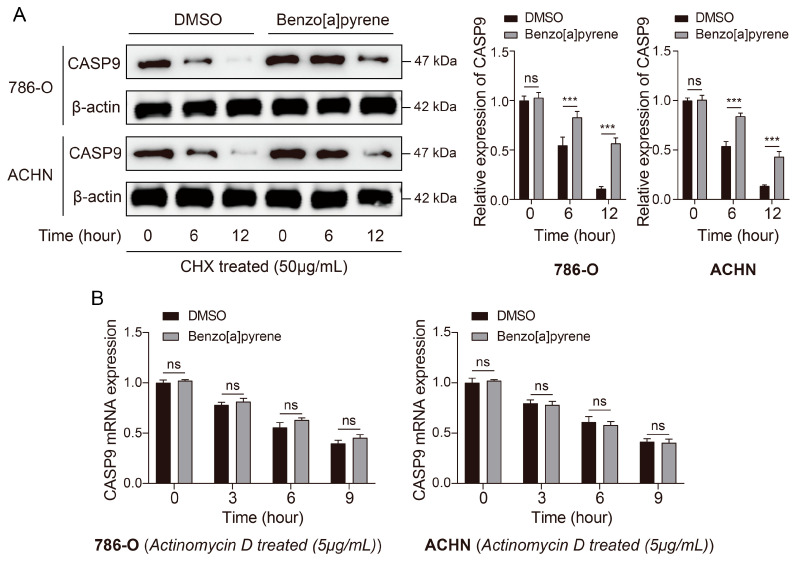
The RCC 786-O and ACHN cells were treated with 0.1 µM BaP for 7 days, and then the protein (**A**) and mRNA (**B**) stability of CASP9 were detected. BaP, benzo[a]pyrene. Values are presented as mean ± SD from at least three independent experiments (n  =  3 replicates); ns, not significant; ***, *p* < 0.001.

**Figure 7:**
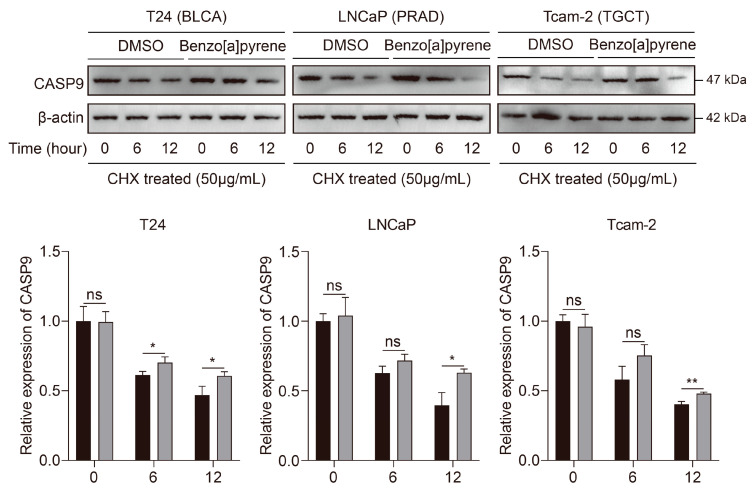
The T24, LNCaP, and Tcam-2 cells were treated with 0.1 µM BaP for 7 days, and then the protein stability of CASP9 was detected. Values are presented as mean ± SD from at least three independent experiments (n  =  3 replicates); ns, not significant; *, *p* < 0.05; **, *p* < 0.01.

On the basis of prior reports indicating that free BaP levels in human serum range from approximately 0.5 nM to 147.3 nM [32], we used 0.1 µM (100 nM) BaP in the present experiments. To mimic the effects of long-term, low-dose exposure, we treated human RCC cell lines 786-O and ACHN with 0.1 µM BaP for 7 days. We then examined whether BaP altered the stability of CASP9 protein and mRNA. The results indicated that chronic BaP exposure significantly enhanced CASP9 protein stability in both RCC cell lines (all *p* < 0.001, Figure 6A). In contrast, BaP did not significantly affect CASP9 mRNA stability (all *p* > 0.05, Figure 6B). To examine the broader relevance of this interaction across urogenital cancers, we treated not only RCC lines (786-O, ACHN) but also representative lines for BLCA (T24), PRAD (LNCaP), and TGCT (Tcam-2) with 0.1 μM BaP for 7 days. Similar to the results in RCC cells, chronic BaP exposure significantly enhanced CASP9 protein stability in T24, LNCaP, and Tcam-2 cells (all *p* < 0.05, Figure 7). The original Western blotting images are provided in Figure S5. Taken together, these findings suggest that BaP binding likely increases CASP9 protein stability at the post-transcriptional level, providing a plausible mechanism through which this common EDC may promote urogenital tumor progression, most likely by modulating apoptotic pathways.

## 4. Discussion

Accumulating evidence indicates that exposure to EDCs is an established environmental factor driving the initiation and progression of urogenital tumors [8,9,10,11]; yet the precise molecular mechanisms and shared targets that connect these phenomena remain incompletely defined. In this study, we adopted an integrated computational and experimental strategy to systematically clarify the pathogenic pathways of 12 prevalent EDCs across four major urogenital malignancies. This analysis, interestingly, revealed a set of key shared protein targets: EGFR and CASP3 in BLCA; EGFR and CASP9 in RCC; CASP3, ESR1, and EGFR in PRAD; and KIT in TGCT. We then chose the representative BaP–CASP9 interaction, because of its strong predicted binding affinity, for experimental validation. Our results showed that chronic exposure to BaP at environmentally relevant levels markedly enhanced CASP9 protein stability in multiple urogenital cancer cells, while leaving mRNA stability unchanged. These findings delineate connections between EDC exposure and urogenital oncogenesis and point to biomarkers and therapeutic targets.

EDCs are widely implicated in cancer risk, yet the molecular logic by which they engage malignant programs across tissue contexts remains poorly resolved. Here, the signal was not diffused. Across urogenital malignancies, EDC-associated targets converged on a restricted set of nodes linked to receptor tyrosine kinase signaling, apoptotic control, steroid receptor activity and, in the germ cell setting, developmental regulation. This degree of convergence is unlikely to reflect network structure alone and instead points to a shared architecture of toxicological vulnerability that is subsequently shaped by lineage. That distinction became apparent when tumor-specific patterns were considered in parallel. In BLCA, EGFR and CASP3 formed the dominant axis, consistent with coordinated perturbation of proliferative signaling and apoptotic execution [33,34]. In RCC, EGFR reappeared. However, the accompanying emergence of CASP9, together with its adverse prognostic association, suggested this apoptotic regulator, similar to CASP5 and PUMA [35,36], may be repurposed in a context-dependent manner. CASP9 would be discussed below. PRAD retained the recurrent EGFR-caspase framework while adding ESR1, placing endocrine responsiveness more centrally in the disease logic [37]. TGCT diverged most clearly from the epithelial tumors, with KIT defining a lineage-restricted developmental signal that is biologically coherent in the germ cell compartment [38,39]. Viewed together, these patterns argue against a single-pathway model of EDC-associated carcinogenesis. Instead, they suggest that endocrine disruptors act on a limited set of conserved signaling liabilities, while tissue identity determines whether those perturbations are interpreted through growth-factor dependence, hormone responsiveness, apoptotic plasticity or developmental instability. Although these inferences remain grounded in computational target prioritization and therefore require direct functional testing, the cross-cancer recurrence of specific nodes indicates that EDC susceptibility is structured rather than random.

The recurrent identification of EGFR across BLCA, RCC, and PRAD is particularly notable. EGFR overexpression is a well-established driver in many epithelial cancers. EDCs may contribute to tumorigenesis by acting as exogenous activators or sensitizers of the EGFR pathway. Certain EDCs, such as Bisphenol A and phthalates, have been reported to induce EGFR transactivation or enhance ligand-dependent EGFR signaling via G-protein coupled receptor crosstalk or reactive oxygen species generation, leading to sustained mitogenic and anti-apoptotic signaling [40,41]. Therefore, chronic EDC exposure could promote tumor progression in susceptible tissues by amplifying signaling through this central oncogenic hub.

EDCs may promote tumor-relevant phenotypes through both canonical endocrine and non-canonical mechanisms. Canonically, EDCs can mimic, antagonize, or otherwise interfere with steroid hormone signaling, including estrogen receptor and androgen receptor pathways. In the present analysis, ESR1 was identified as a hub target in prostate adenocarcinoma, suggesting that estrogen receptor-related signaling may represent one route through which EDCs influence urogenital tumor biology. Although AR is highly relevant to prostate cancer biology, it did not emerge as a shared hub target under the filtering strategy used in this study. Beyond classical steroid receptors, EDCs may also act through receptor tyrosine kinase signaling, mitochondrial apoptosis, oxidative stress, DNA damage responses, and xenobiotic metabolism pathways. The identification of EGFR, CASP3, CASP9, and KIT is consistent with these non-canonical mechanisms. In particular, BaP is known to act as a polycyclic aromatic hydrocarbon with genotoxic and xenobiotic-response properties, and its predicted and experimentally supported interaction with CASP9 suggests a possible link between environmental exposure and apoptosis-related regulation in RCC cells.

BaP was selected for experimental validation based on an integrated prioritization strategy rather than because it was considered the only relevant EDC. Specifically, BaP showed consistent carcinogenic potential across multiple prediction platforms, strong predicted binding affinity toward CASP9, favorable binding stability in molecular dynamics simulations, and biological relevance because CASP9 was identified as a hub target in RCC with prognostic significance. In addition, BaP is a widely distributed environmental polycyclic aromatic hydrocarbon with well-established carcinogenic properties. Thus, the BaP–CASP9 axis was chosen as a representative proof-of-concept interaction for experimental validation, while other EDC–target combinations remain important candidates for future investigation. In RCC cells, BaP exposure increased CASP9 protein stability without altering CASP9 mRNA stability, suggesting post-transcriptional regulation. These data provide experimental support for the computationally predicted BaP-CASP9 interaction. This stabilization of CASP9, a well-established initiator of the intrinsic apoptotic cascade, seems paradoxical at first glance. Still, it aligns with both its adverse prognostic association and growing evidence that caspases may also exert non-canonical, pro-tumorigenic functions in RCC [35,36]. Recent spatial transcriptomic findings may help make sense of this contradiction, showing that CASP9-high RCC cells define a spatially organized, immunosuppressive state. These cells are located near macrophage-enriched regions and appear to participate in pro-tumor crosstalk, plausibly mediated by signaling axes such as SPP1-CD44. The consequence is an immunosuppressive tumor microenvironment that supports RCC progression [42]. Therefore, our findings suggest that BaP-mediated CASP9 stabilization may divert CASP9 from its conventional apoptotic role and instead support an alternative route by which environmental EDCs contribute to renal carcinogenesis. However, the current results should be interpreted as evidence of mechanistic plausibility rather than direct proof that BaP exposure causes RCC in humans.

The identification of shared molecular targets, like CASP9 in RCC and EGFR in cancers, creates translational opportunities. These proteins could be biomarkers for estimating environmental risk and enabling earlier tumor detection. Assessing CASP9 expression in exposed individuals may refine RCC risk stratification. Previous study has proved that functional polymorphisms of CASP9 served as a biomarker for the occurrence of RCC [43]. These hubs are also potential therapeutic targets in EDC-associated cancers. Long-term EDC exposure may induce EGFR and CASP9, so targeted strategies like EGFR inhibitors, such as Osimertinib and Lazertinib [44], or compounds to reestablish apoptotic signaling should be considered. The molecular links strengthen the rationale for regulating EDCs, which is relevant to public health. Distinguishing correlation from causation remains crucial for prevention strategies. The ultimate goal of such efforts is to limit exposure to widespread environmental carcinogens and, over time, reduce the burden of urogenital malignancies.

This study has several limitations. First, the integrated strategy remains largely predictive, and further experimental validation of prioritized EDC–target interactions across all four cancers is needed. Second, the 12 EDCs studied are not exhaustive. Third, although BaP stabilizes CASP9 post-transcriptionally, the exact mechanism requires clarification using biochemical and degradation-pathway assays. Moreover, direct binding of BaP to CASP9, while supported computationally, still lacks definitive biochemical proof. Future studies should directly test whether BaP suppresses the ubiquitin–proteasome-mediated turnover of CASP9. Specifically, proteasomal inhibition assays combined with cycloheximide chase experiments, as well as ubiquitination pulldown assays comparing BaP-treated versus control cells, would clarify whether BaP enhances CASP9 stability by reducing its ubiquitination and subsequent proteasomal degradation. Fourth, IHC relied on public database images; validation with well-annotated clinical samples is warranted. The absence of patient-level BaP exposure data also precludes direct correlation with tumor expression or outcomes. Fifth, sex-specific differences in EDC susceptibility were not fully addressed; future studies should incorporate sex-stratified cohorts and models.

## 5. Conclusions

In conclusion, our integrated computational analysis identified a landscape of shared molecular targets linking 12 major EDCs with 4 principal urogenital cancers. We identified and computationally validated a group of high-confidence hub targets, including EGFR and CASP3 in BLCA, EGFR and CASP9 in RCC, CASP3, ESR1, and EGFR in PRAD, and KIT in TGCT, which may serve as critical bridges between environmental exposure and oncogenesis. Focused experimental validation demonstrated that the prioritized BaP–CASP9 interaction functionally stabilizes CASP9 protein across urogenital cancer cell lines, providing proof-of-concept for the pipeline. These results help clarify potential converging pathways through which distinct EDCs may perturb endocrine-related, apoptotic, receptor tyrosine kinase, and developmental signaling networks associated with urogenital tumor biology. While the present findings support mechanistic plausibility, further exposure-based clinical and experimental studies are needed to establish causal relationships between specific EDCs and urogenital tumor development.

## Data Availability

Data will be made available on request.

## Supplementary Materials

The following supporting information can be downloaded at: https://www.mdpi.com/article/10.3390/biology15120946/s1. Table S1. Molecular formula and SMILES structure of EDCs. Table S2. The keywords for identifying urogenital tumors-related genes. Table S3. The PDB IDs of hub targets linking EDCs to urogenital tumors. Table S4. The primer sequences used in this study. Table S5. The public databases used in this study. Figure S1. Sensitivity analysis of the target prediction. Figure S2. The shared targets of EDCs in RCC. Figure S3. The shared targets of EDCs in PRAD. Figure S4. The shared targets of EDCs in TGCT. Figure S5. The original Western blotting images.
