# Supplementary material for: Investigating the Shared Mechanisms of Endocrine-Disrupting Chemicals in Urogenital Tumors
Source: Biology (Basel). 2026 Jun 17;15(12):946. doi: 10.3390/biology15120946 (PMC13295656; doi:10.3390/biology15120946)
Supplement: Supplementary file 1 [file biology-15-00946-s001.zip › biology-4303675-Supplementary Figures.pdf]

Supplementary Figures

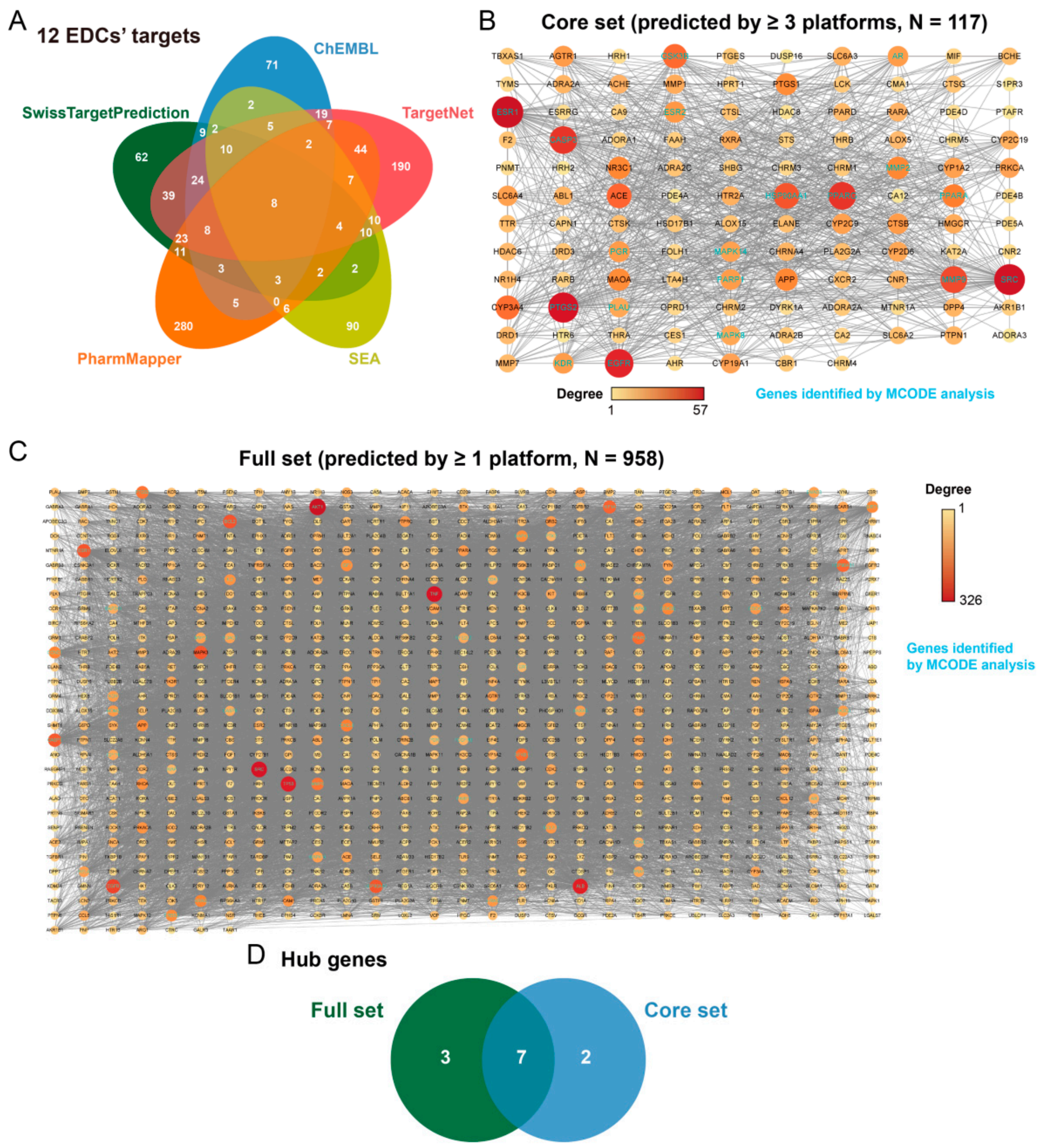

Fig. S1. Sensitivity analysis of the target prediction. **A** A venn plot indicating the intersections of the targets predicted by ChEMBL, PharmMapper, Similarity Ensemble Approach (SEA), SwissTargetPrediction, and TargetNet. **B** A core set of 117 targets (those predicted by ≥ 3 platforms) of EDCs was established, and 9 hub targets were identified by cytoHubba analysis and MCODE analyses. **C** A full set of 958 targets (those predicted by ≥ 1 platforms) of EDCs was established, and 10 hub

targets were identified by cytoHubba analysis and MCODE analyses. **D** PTGS2, CASP3, SRC, PPARG, EGFR, ESR1, and HSP90AA1 remained consistent between the two analyses. *EDC, endocrine-disrupting chemicals.*

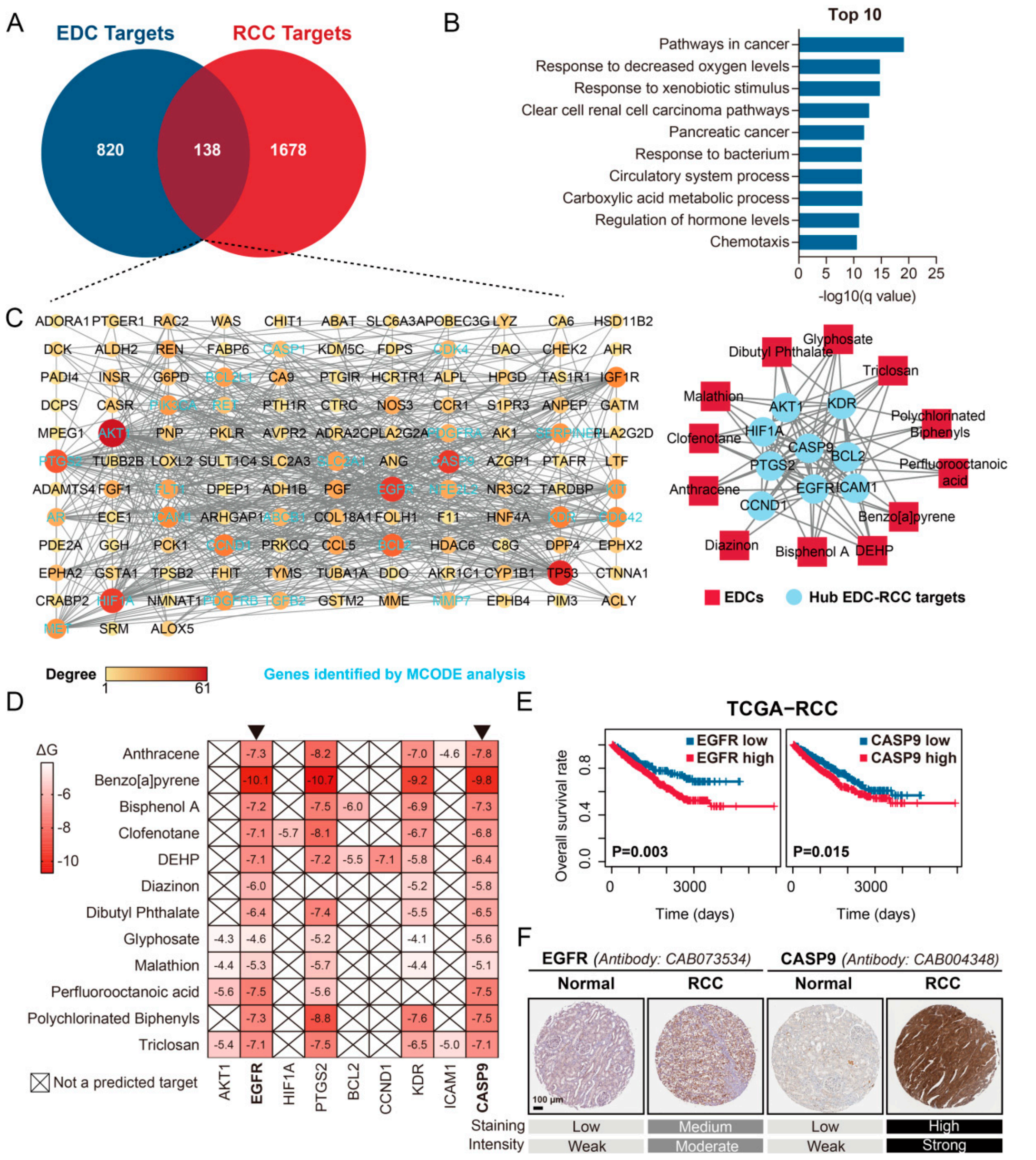

Fig. S2. The shared targets of EDCs in RCC. **A** 138 proteins were identified as the potential EDC-RCC targets. **B** Functional enrichment analysis of the 138 EDC-RCC targets performed using Metascape. **C** The PPI network analysis identified 9 hub

genes, and EGFR and CASP9 served as shared targets of all 12 EDCs in RCC. **D** Molecular docking and molecular dynamics simulation indicated the binding affinity of the EDCs with their targets. **E** The prognosis value of EGFR and CASP9 in TCGA-RCC cohort, the optimal cut-off values were detected by the X-tile software. **F** IHC analyses indicated the expression levels of EGFR and CASP9 in normal kidney and RCC tissues. *RCC, renal cell carcinoma; PPI, protein-protein interaction; TCGA, the cancer genome atlas; IHC, immunohistochemistry.*

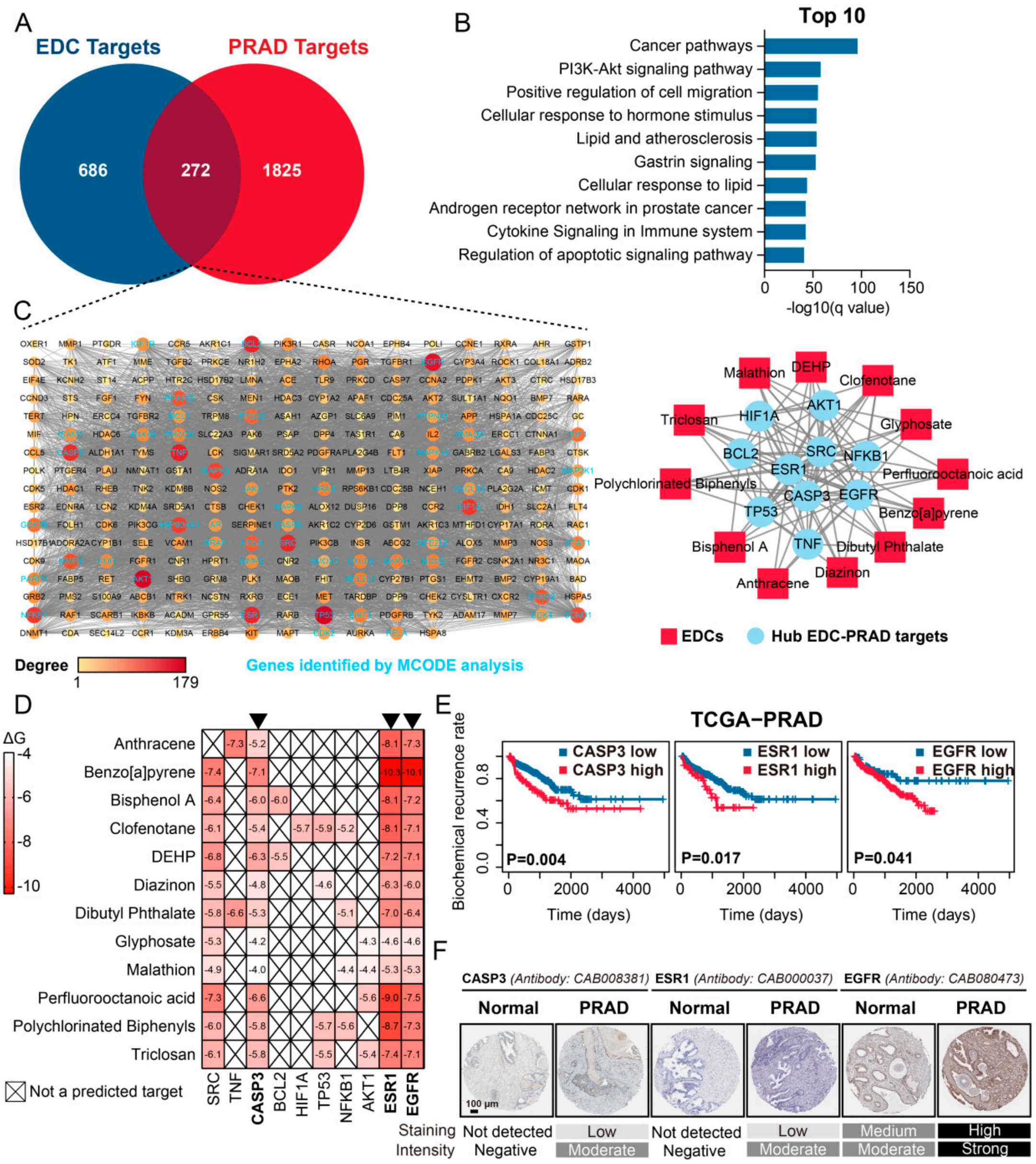

Fig. S3. The shared targets of EDCs in PRAD. **A** 272 proteins were identified as the potential EDC-PRAD targets. **B** Functional enrichment analysis of the 272 EDC-PRAD targets performed using Metascape. **C** The PPI network analysis identified 10 hub genes, and CASP3, ESR1, and EGFR served as shared targets of all 12 EDCs in PRAD. **D** Molecular docking and molecular dynamics simulation indicated the binding affinity of the EDCs with their targets. **E** The prognosis value of CASP3, ESR1, and EGFR in TCGA-PRAD cohort, the optimal cut-off values were

detected by the X-tile software. **F** IHC analyses indicated the expression levels of CASP3, ESR1, and EGFR in normal prostate and PRAD tissues. *PRAD, prostate cancer.*

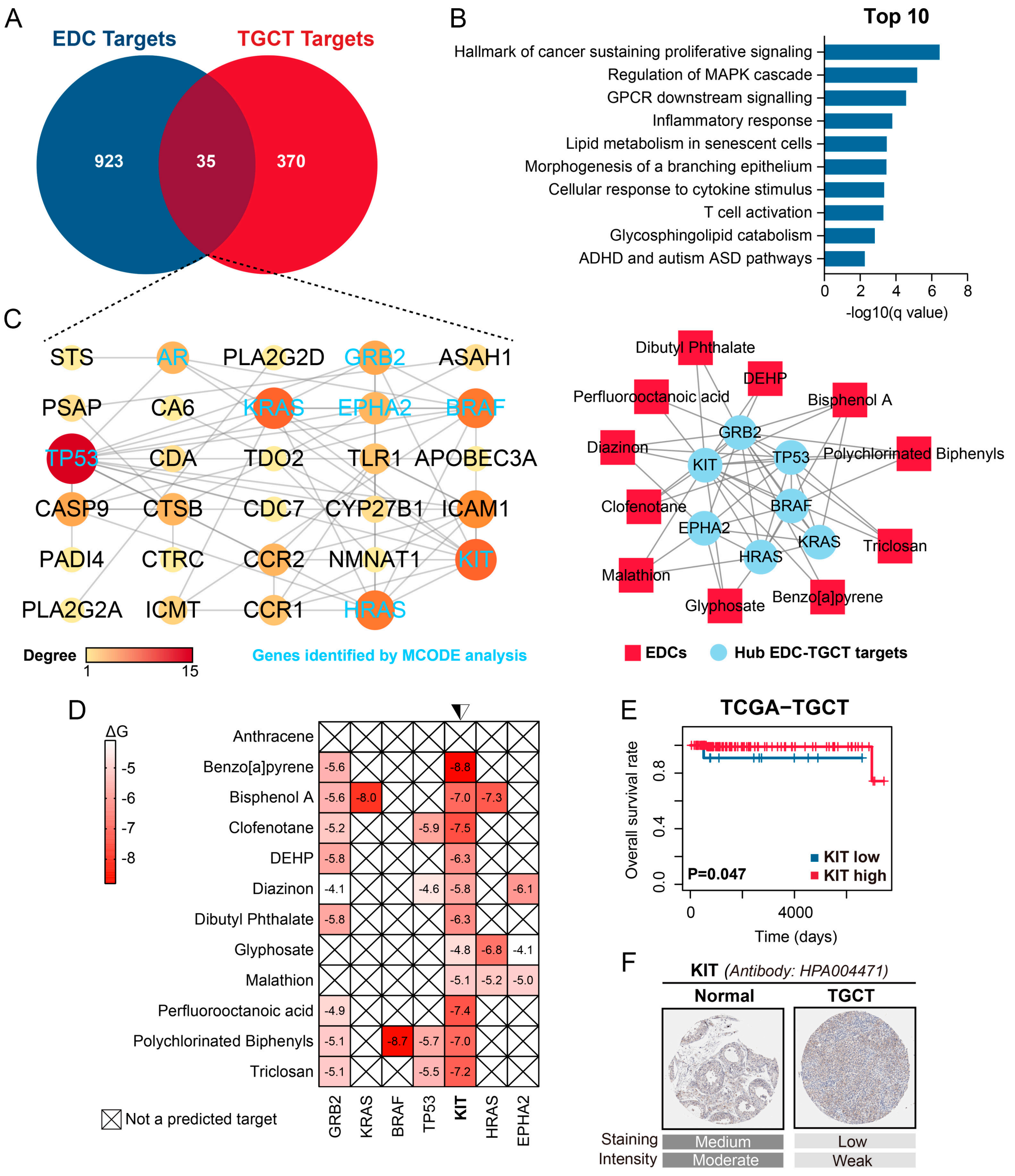

Fig. S4. The shared targets of EDCs in TGCT. **A** 35 proteins were identified as the potential EDC-TGCT targets. **B** Functional enrichment analysis of the 35 EDC-TGCT targets performed using Metascape. **C** The PPI network analysis identified 7 hub

genes. KIT served as a shared target for 11 of the 12 EDCs, with anthracene as the only exception. **D** Molecular docking and molecular dynamics simulation indicated the binding affinity of the EDCs with their targets. **E** The prognosis value of KIT in TCGA-TGCT cohort, the optimal cut-off values were detected by the X-tile software. **F** IHC analyses indicated the expression levels of KIT in normal testis and TGCT tissues. *TGCT, testicular germ cell tumor*.

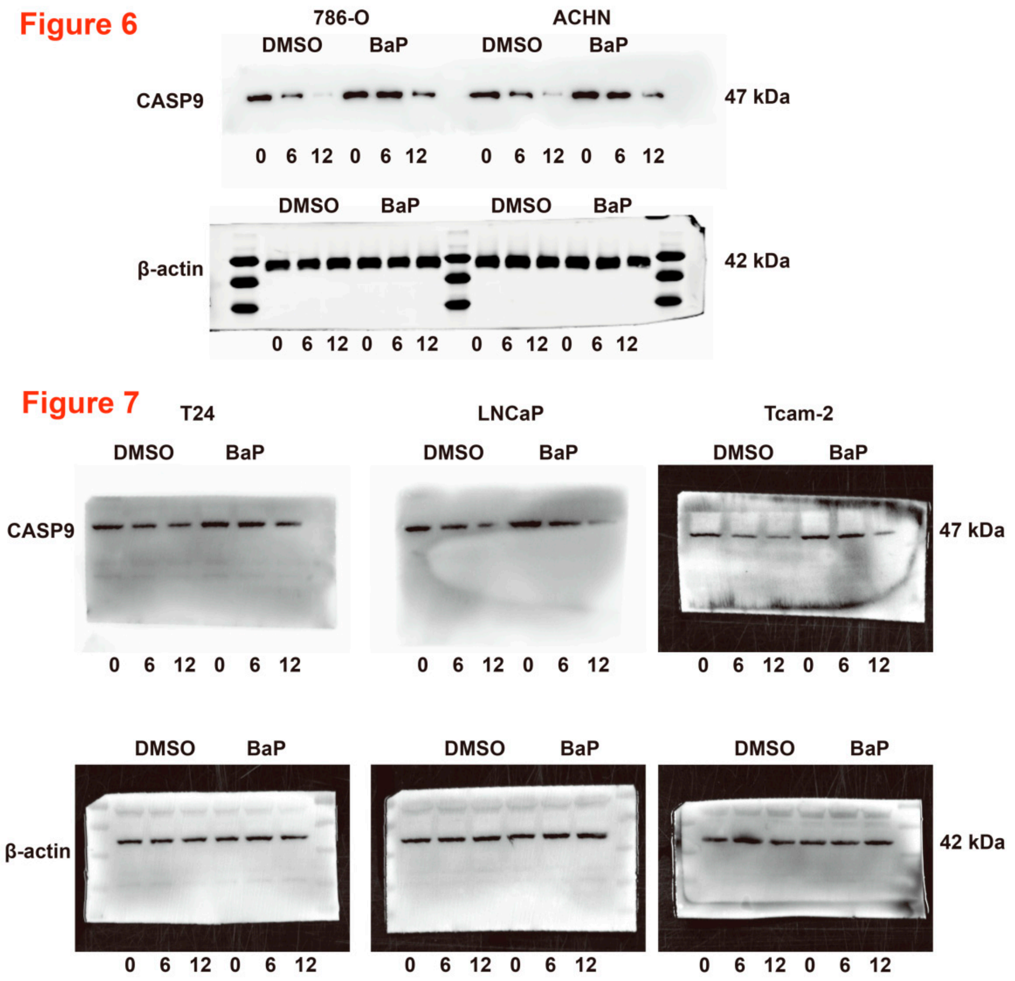

Fig. S5. The original western blotting images.
