# Supplementary material for: Investigating the Shared Mechanisms of Endocrine-Disrupting Chemicals in Urogenital Tumors
Source: Biology (Basel). 2026 Jun 17;15(12):946. doi: 10.3390/biology15120946 (PMC13295656; doi:10.3390/biology15120946)
Supplement: Supplementary file 1 [file biology-15-00946-s001.zip › biology-4303675-Supplementary Tables.pdf]

Supplementary Tables

Table S1 Molecular formula and SMILES structure of EDCs.

EDCs	Molecular formulas	SMILES structures
Anthracene	C14H10	C1=CC=C2C=C3C=CC=CC3=CC2=C1
Benzo[a]pyrene	C20H12	C1=CC=C2C3=C4C(=CC2=C1)C=CC5=C4C(=CC=C5)C=C3
Bisphenol A	C15H16O2	CC(C)(C1=CC=C(C=C1)O)C2=CC=C(C=C2)O
Clofenotane	C14H9Cl5	C1=CC(=CC=C1C(C2=CC=C(C=C2)Cl)C(Cl)(Cl)Cl)Cl
DEHP	C24H38O4	CCCCC(CC)COC(=O)C1=CC=CC=C1C(=O)OCC(CC)CCCC
Diazinon	C12H21N2O3PS	CCOP(=S)(OCC)OC1=NC(=NC(=C1)C)C(C)C
Dibutyl Phthalate	C16H22O4	CCCCOC(=O)C1=CC=CC=C1C(=O)OCCCC
Glyphosate	C3H8NO5P	C(C(=O)O)NCP(=O)(O)O
Malathion	C10H19O6PS2	CCOC(=O)CC(C(=O)OCC)SP(=S)(OC)OC
Perfluorooctanoic acid	C8HF15O2	C(=O)(C(C(C(C(C(C(C(F)(F)F)(F)F)(F)F)(F)F)(F)F)(F)F)(F)F)O
Polychlorinated Biphenyls	C12H4Cl6	C1=C(C(=CC(=C1Cl)Cl)Cl)C2=CC(=C(C=C2Cl)Cl)Cl
Triclosan	C12H7Cl3O2	C1=CC(=C(C=C1Cl)O)OC2=C(C=C(C=C2)Cl)Cl

Table S2 The keywords for identifying urogenital tumors-related genes

Urogenital tumors	Abbreviation	Keywords
Bladder cancer	BLCA	Bladder Cancer
		Bladder Carcinoma
		Malignant Neoplasm of Bladder
		Urothelial Carcinoma of Bladder
Renal cell carcinoma	RCC	Renal Cell Carcinoma
		Renal Cell Cancer
		Renal Cell Adenocarcinoma
		Renal Cancer
		Kidney Cancer
		Kidney Chromophobe
		Chromophobe Renal Cell Carcinoma
		Renal Chromophobe Cell Carcinoma
		Chromophobe Carcinoma of Kidney
		Kidney Renal Clear Cell Carcinoma
		Clear Cell Renal Cell Carcinoma
		Clear Cell Carcinoma of Kidney
		Clear Cell Kidney Cancer
		Kidney Renal Papillary Cell Carcinoma
		Papillary Renal Cell Carcinoma
		Papillary Kidney Cancer
Prostate cancer	PRAD	Prostate Adenocarcinoma
		Prostate Cancer

		Adenocarcinoma of Prostate<br>Prostate Carcinoma<br>Prostatic Adenocarcinoma<br>Prostatic Carcinoma<br>Prostatic Cancer
Testicular germ cell tumor	TGCT	Testicular Germ Cell Tumor<br>Germ Cell Tumor of Testis<br>Seminoma<br>Non-seminomatous Germ Cell Tumor

Table S3 The PDB IDs of hub targets linking EDCs to urogenital tumors.

Gene Symbol	PDB ID
TP53	1C26
EGFR	8F1X
CASP3	2C1E
ESR1	1XPC
PTGS2	5F19
AKT1	1H10
ERBB2	7PCD
TNF	2ZPX
KRAS	4DSN
HIF1A	4H6J
BCL2	2W3L

CCND1	2W96
EPHA2	1MQB
CASP9	1JXQ
KDR	1YWN
ICAM1	1IAM
SRC	1A07
NFKB1	1SVC
GRB2	1JYR
BRAF	1UWH
KIT	1T45
HRAS	121P

Table S4 The primer sequences used in this study.

**Gene symbol**	**Sequences (5'-3')**	**Amplicon Size (bp)**	**Tm (° C)**	**PCR Efficiency (%)**
CASP9	F: CTTCGTTTCTGCGAACTAACAGG	75	61.6	89.68%
	R: GCACCACTGGGGTAAGGTTT		62.1	
GAPDH	F: GGAGCGAGATCCCTCCAAAAT	197	61.6	97.04%
	R: GGCTGTTGTCATACTTCTCATGG		60.9	

Table S5 The public databases used in this study

**Database**	**Website links**
PubChem	https://pubchem.ncbi.nlm.nih.gov/
admetSAR 3.0	https://lmmd.ecust.edu.cn/admetsar3/index.php
ProTox 3.0	https://tox.charite.de/protox3/index.php?site=home
ADMETlab 3.0	https://admetlab3.scbdd.com/
ChEMBL	https://www.ebi.ac.uk/chembl/
PharmMapper	http://www.lilab-ecust.cn:81/pharmmapper/
SEA	https://sea.bkslab.org/
SwissTargetPrediction	https://www.swisstargetprediction.ch/
TargetNet	http://targetnet.scbdd.com/
UCSC Xena	https://xenabrowser.net/datapages/
TTD	https://ttd.idrblab.cn/
OMIM	https://omim.org/
STRING	https://cn.string-db.org/
Metascape	https://metascape.org/gp/index.html#/main/step1
